# Astrocytes in the ventral pallidum extinguish heroin seeking through GAT-3 upregulation and morphological plasticity at D1-MSN terminals

**DOI:** 10.1038/s41380-021-01333-5

**Published:** 2021-10-12

**Authors:** Anna Kruyer, Danielle Dixon, Ariana Angelis, Davide Amato, Peter W. Kalivas

**Affiliations:** 1 Department of Neuroscience, Medical University of South Carolina, Charleston, SC USA; 2 Department of Psychiatry and Psychotherapy, Charité - Universitätsmedizin Berlin, Berlin, Germany

**Keywords:** Neuroscience, Psychology

## Abstract

GABAergic projections from the nucleus accumbens core to the dorsolateral ventral pallidum are necessary for drug-conditioned cues to initiate relapse-like drug seeking. Astrocytes in the ventral pallidum are situated perisynaptically and regulate GABA transmission through expression of GABA uptake transporters, but whether they are involved in regulating drug seeking is unknown. To determine the contribution of ventral pallidal astrocytes to heroin seeking, we labeled astrocytes in male and female rats with a membrane-bound fluorescent tag and used confocal microscopy to quantify astroglial expression of the GABA transporter GAT-3 and astrocyte synaptic proximity after withdrawal from heroin self-administration and during 15 min of cued heroin seeking. We found that GAT-3 was upregulated in rats that had extinguished heroin seeking, but not in animals that were withdrawn from heroin without extinction training or in rats that extinguished sucrose seeking. When GAT-3 upregulation was reversed using a vivo-morpholino oligo, heroin seeking was restored in the extinguished context and extinction of cued heroin seeking was disrupted compared to control animals. Although astrocyte synaptic proximity was not altered overall after heroin withdrawal, examination of astrocyte proximity to accumbens D1- or D2-expressing afferents revealed a selective increase in astrocyte proximity with D1-expressing terminals during extinction of heroin self-administration. Experimentally-induced reduction of astrocyte synaptic proximity through knockdown of the astrocyte-selective actin-binding protein ezrin also markedly disrupted extinction of heroin seeking. Notably, GAT-3 or ezrin knockdown had no impact on context- or cue-induced seeking in sucrose-trained animals. These data show that astrocytes in the ventral pallidum undergo plasticity after extinction of heroin use that reduces seeking and highlight the importance of astrocyte-neuron interactions in shaping behaviors associated with opioid use disorder.

## Introduction

Relapse vulnerability is a principal feature of opioid use disorder [1, 2] and activity in GABAergic projections from the nucleus accumbens core (NAcore) to the dorsolateral ventral pallidum (dlVP) is necessary for drug-associated cues to elicit drug seeking in rodent models of relapse [3, 4]. This pathway is an important regulator of both natural reward and drug seeking [5], but in contrast with natural rewards, repeated use of addictive drugs causes dysregulation of glutamate homeostasis in the NAcore [6]. The resulting changes in excitatory transmission onto D1 and D2 receptor-expressing medium spiny neurons (D1- and D2-MSNs) result in divergent synaptic adaptations that uniquely impact transmitter release in the dlVP [7, 8] and produce drug-seeking behaviors [9–11]. For instance, after extinction from cocaine self-administration, D2-MSNs undergo enkephalin-dependent long-term depression not observed at D1-MSN terminals [7]. In general, it has been concluded that D1-MSN activity within this circuit drives cued drug seeking, while D2-MSN transmission opposes it, perhaps by facilitating extinction [12–14]. Similarly, distinct GABAergic and glutamatergic neuron subpopulations in the dlVP promote or inhibit drug seeking, respectively [8, 15, 16].

While much has been learned about how neural plasticity in the NAcore to dlVP circuit regulates drug seeking, the discovery process for drug-induced plasticity has largely ignored the critical regulation of synaptic signaling by astroglia. Astroglia promote synaptogenesis during brain development and after injury by secreting synaptogenic proteins, such as thrombospondin [17–19]. Astroglia also markedly affect ongoing synaptic transmission in the adult rodent brain through changes in surface expression of transporters that remove synaptically released glutamate and GABA [20–22] and through their own structural plasticity, which impacts synaptic proximity of transporters expressed on astroglial peripheral processes [23–25]. Repeated drug use engages these two mechanisms of astroglial plasticity to modulate glutamate transmission in the NAcore and promotes drug seeking by reducing the expression of GLT-1 [26, 27] and causing enduring retraction of astroglial processes that insulate synapses [27]. In contrast, a role for astroglial modulation of GABAergic transmission in drug-induced plasticity has not been studied.

The dlVP is a recipient of dense GABAergic innervation from both D1- and D2-MSNs in the NAcore [28] and dlVP astrocytes express the two main GABA transporter subtypes, GAT-1 and GAT-3, that terminate GABAergic synaptic transmission and regulate GABA spillover [29]. Of these two, GAT-3 is expressed exclusively by astrocytes and is found largely on astroglial processes that serve to modulate tonic inhibitory currents in post-synaptic cells [22, 30, 31]. We hypothesized that the proximity of astrocyte processes to synapses in the dlVP and the expression of GAT-3 would undergo long-lasting adaptations after withdrawal from heroin self-administration and transient plasticity in response to heroin-associated cues that trigger heroin seeking. We also hypothesized that these astroglial adaptations would occur in response to heroin use, but not in rats trained to self-administer sucrose, a natural reward. To test these hypotheses, we labeled dlVP astroglia in rats that were trained to self-administer heroin or sucrose. After withdrawal, with or without extinction training or during 15 min of cued reinstatement, we quantified expression of GAT-3 on individual astrocytes in the dlVP, and the co-registration of the astroglial membrane with the presynaptic marker synaptojanin 1 (SYNJ1) using confocal microscopy [32]. We found that extinction of heroin use increased GAT-3 expression and elevated astrocyte proximity to D1-expressing synapses. Moreover, cued heroin seeking was associated with a partial reversal of the extinction-induced increases in both GAT-3 and astrocyte proximity to D1-expressing synapses in the dlVP. To determine the impact of these astroglial adaptations on heroin seeking behavior, we delivered vivo-morpholino oligomers against GAT-3 or ezrin, an astrocyte-specific actin-binding protein that contributes to astrocyte peripheral process elongation [25], prior to cued reinstatement. We found that preventing either adaptation in dlVP astroglia reversed extinction training and prolonged cue-induced seeking in heroin-trained animals, but did not impact context- or cue-induced seeking in sucrose-trained rats. Our findings demonstrate that astrocytes in the dlVP undergo subcircuit-selective morphological plasticity and changes in transporter expression that critically control the extinction of heroin seeking.

## Methods

### Self-administration

Animal procedures were approved by the Institutional Animal Care and Use Committee at the Medical University of South Carolina. Male and female Drd1a-iCre and Drd2-iCre (D1- and D2-Cre) rats (National Institute on Drug Abuse) and their wild-type littermates were bred in house and maintained on a 12-h reverse light/dark cycle. Rats were caged with littermates until surgery, after which they were singly housed. When animals reached 200–250 g they were anesthetized with ketamine/xylazine and fitted with intrajugular catheters (Instech Laboratories) that were flushed daily with taurolidine-citrate catheter lock solution (Access Technologies) to maintain patency. Animals undergoing operant training with sucrose received no catheters. After recovery, animals were trained to self-administer heroin during 3 h sessions for 10 days and active lever presses resulted in i.v. heroin infusions (100 μg/infusion for sessions 1–2, 50 μg/infusion for sessions 3–4, 25 μg/infusion for sessions 5–10) and light/tone cues for 5 s. Sucrose animals self-administered unflavored sucrose pellets (45 mg, Bio-Serv) for oral consumption during 2 h daily sessions for 10 days. Yoked saline animals received i.v. saline along with cues when a paired rat self-administered heroin. Yoked controls for sucrose-trained animals received cues, but no pellet when a paired rat self-administered sucrose. All animals received 25 g chow/day throughout self-administration. Animals next underwent 10–11 days of extinction training (3 h/day for heroin-trained rats or 2 h/day for sucrose-trained rats) during which time active lever pressing had no consequence. Extinguished rats and yoked controls were sacrificed 48 h after the last extinction session. Rats in the abstinent group were sacrificed after a corresponding time of abstinence without extinction training. Reinstated rats were placed in the operant chamber a final time for 15 or 120 min and light/tone cues were restored to the active lever, but no reward was delivered.

### Viral labeling

**Fig. 1:**
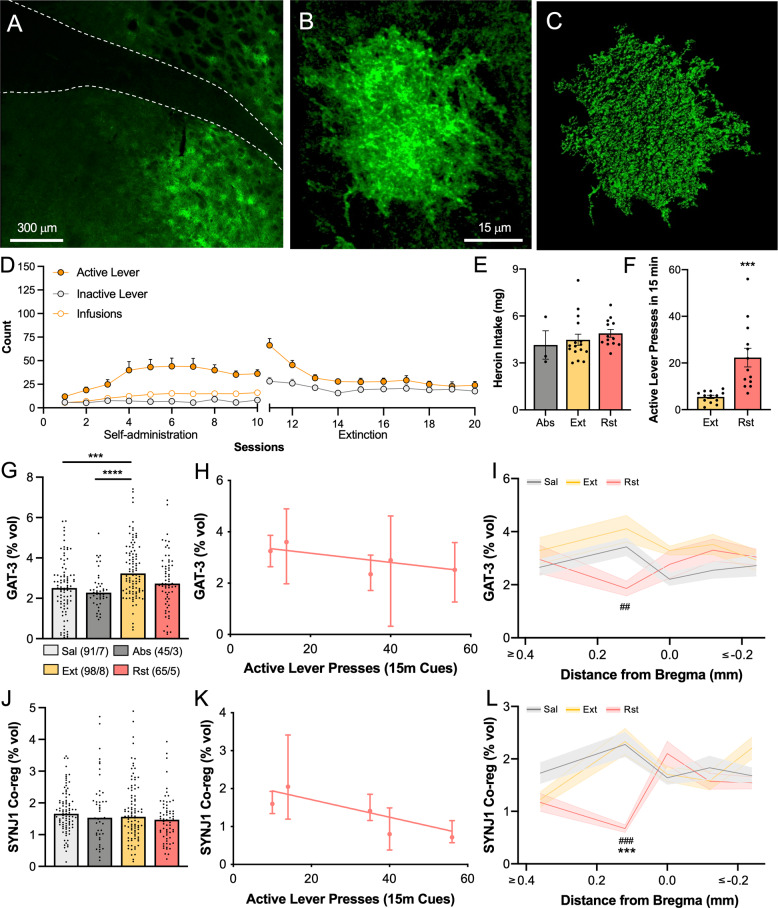
Astrocytes in the dlVP upregulate GAT-3 after extinction of heroin use. **A** Astroglia in the dlVP were labeled using AAV5.gfaABC1D.Lck.GFP. Raw signal **B** and digitized render **C** of an astrocyte from a yoked control animal expressing membrane-targeted GFP. **D** Rats underwent heroin self-administration and extinction training and heroin intake was not different between heroin-treated groups (**E**, one-way ANOVA *F*(2,29) = 0.2356, *p* = 0.7916). **F** Reinstated animals were exposed to heroin-associated cues for 15 min and active lever pressing increased relative to lever pressing during the first 15 min of extinction training 48 h prior (Student’s *t*(12) = 4.470, ***p = 0.0008). **G** Immunofluorescent labeling of the astroglial GABA transporter GAT-3 revealed upregulated expression after heroin extinction relative to saline controls and animals that self-administered heroin, but did not undergo extinction training (Kruskal–Wallis = 26.65, *p* < 0.0001, ***p = 0.0002, ****p < 0.0001 using Dunn’s post hoc test). **H** Astroglial GAT-3 expression correlated negatively with active lever pressing in animals that underwent cued reinstatement (Spearman’s *r* = –0.900, *p* = 0.04). **I** When GAT-3 expression was analyzed according to stereotaxic coordinates of imaged astroglia, a reduction in GAT-3 expression was noted at +0.12 mm from bregma along the anterior-posterior axis in reinstated animals (two-way ANOVA treatment *F*(2,221) = 3.819, *p* = 0.0234, ^##^*p* = 0.0068 Ext v. Rst). Although unchanged when all dlVP astroglia were analyzed together (**J**, Kruskal–Wallis = 5.576, *p* = 0.1342), astroglial co-registration with the presynaptic marker synaptojanin 1 (SYNJ1) correlated negatively with active lever pressing in reinstated animals (**K**, Spearman’s *r* = –0.900, *p* = 0.04). SYNJ1 co-registration by dlVP astroglia was reduced during 15 min of heroin cue exposure at +0.12 mm from bregma along the anterior-posterior axis (**L**, two-way ANOVA treatment *F*(2,222) = 5.393, *p* = 0.0052, ***p = 0.002 Sal v. Rst, ^####^*p* < 0.001 Ext v. Rst). Data shown as mean ± SEM (**D**–**F**, **I**, **L**), median (**G**, **J**), or median ± 95% CI (**H**, **K**). *N* shown in legend as cells/animals (**G**, **J**). Sal yoked saline, Abs abstinence, Ext extinction, Rst 15-min reinstatement.

Following intrajugular catheter placement or 5 days prior to the beginning of operant sucrose training, AAV5.gfaABC1D.Lck-GFP (Addgene) was microinjected (0.7 μL, 0.15 μL/min, 5 min diffusion) bilaterally in the dlVP (+0.0 mm AP, ±2.2 mm ML, –7.4 mm DV; Fig. 1A). For terminal-specific co-registration studies, AAV2.hSyn.DIO.hM3d-mCherry (Addgene) was also microinjected (1 μL, 0.15 μL/min, 5 min diffusion) bilaterally in the NAcore (+1.5 mm AP, ±1.4 mm ML, –6.6 mm DV) of D1- and D2-Cre rats.

### Immunohistochemistry

Immediately following 15 min of cued reinstatement or 48 h after the last extinction session or after a corresponding period of abstinence, rats received an intrahepatic injection of ketamine (100 mg/kg) before undergoing perfusion with 60 mL 1X phosphate buffer and 120 mL 4% paraformaldehyde (EM Sciences). Brains were extracted and submerged in 4% paraformaldehyde for 24 h at 4 °C before transfer into 1X phosphate buffer. Brains were sliced at 50 μm using a vibrating blade microtome (Leica) and slices were stored in a glycerol-based storage solution. For staining, sections containing the dlVP were permeabilized for 15 min at room temperature using 2% Triton X-100 in 1X phosphate-buffered saline. Tissue was transferred into block containing 0.2% Triton X-100 and 2% normal goat serum and incubated in primary antibodies (mouse anti-SYNJ1, BD Biosciences #612248 and rabbit anti-GAT-3, Abcam ab181783) at 1:1000 in block for 24 h at 4 °C. The monoclonal anti-SYNJ1 antibody was previously validated by western blot and detects only one band in adult rat brain at ~145 kDa, the estimated molecular weight of SYNJ1 [33]. GAT-3 immunoreactivity shown in Supplementary Fig. S1 corresponds to the pattern of GAT-3 expression identified using in situ hybridization in mouse [34]. Tissue was washed in 1X phosphate-buffered saline with 0.2% Triton X-100 and incubated in Alexa Fluor-conjugated secondary antibodies (Invitrogen, 1:1000) for 24 h at room temperature. Tissue was then washed and mounted onto glass slides and coverslipped using ProLong Gold mountant (Thermo Fisher). This staining protocol yields consistent tissue penetration of SYNJ1 and GAT-3 immunoreactivity in 50 μm tissue (Supplementary Fig. S2).

### Confocal microscopy and image analysis

**Fig. 2:**
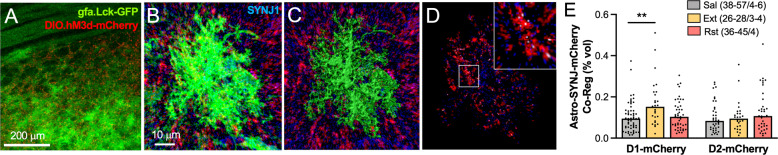
Morphological plasticity of dlVP astroglia was associated with D1-MSN terminals from the NAcore. **A** dlVP astroglia (green) were co-labeled with terminals from NAcore D1- or D2-MSNs (red). Imaged astrocytes (green, **B**) were digitally rendered (**C**) and their co-registration with mCherry-labeled terminals (red) and SYNJ1 (blue) was quantified (**D**, white; box magnified in inset). **E** The degree of triple co-registration in D1-Cre rats was elevated during extinction training, indicating increased astroglial proximity to synapses containing D1-MSN terminals (Kruskal–Wallis = 10.38, *p* = 0.0056, **p = 0.004 Ext v. Sal using Dunn’s test). This increase was restored to control levels during cued reinstatement (*p* > 0.9999 Sal v. Rst using Dunn’s test). No changes were detected at synapses containing D2-MSN terminals (**D**, Kruskal–Wallis = 1.571, *p* = 0.4560). **E** Data shown as median, *N* shown in legend as cells/animals. Sal yoked saline, Ext extinguished, Rst 15-min reinstated.

Astroglia were imaged with a 63× oil immersion objective lens using a Leica SP5 confocal microscope. Z-series containing 3–8 astroglia were imaged at 12-bit resolution with a pinhole of 1 Airy unit, a frame size of 1024 × 1024, two-line averaging and a 1-μm step between *z*-planes and *z*-stacks were deconvolved ten times (Auto-Quant). Animals were given alphanumerical designations following behavioral studies that were maintained throughout image analysis, so that all analyses were conducted by an investigator blind to animal treatment. Imaging parameters were kept constant for each marker analyzed and experimental groups were stained and imaged concurrently for each analysis. During imaging, distance from bregma was first determined by comparing the anterior commissure shape and position with atlas figures [35]. The subregion of the dorsal ventral pallidum that was both subcommissural and lateral to the ventricle was considered to be dorsolateral, and not ventromedial, ventral pallidum (as in [36–39] and Fig. 1A and Supplementary Fig. S1). After initial analysis revealed strong effects of reinstatement at +0.12 mm AP (Fig. 1), subsequent analyses were restricted proximal to this portion of the dlVP. Astrocytes were identified based on characteristic morphology and GFP signal intensity relative to noise and digitally rendered to obtain a metric of astroglial volume (Fig. 1B, C, Bitplane Imaris). Since imaged stacks were maximally 50 μm due to slice thickness, astroglial volume was not reported given likelihood of cropping across *z*, but was instead used to normalize co-registered signal. Astrocyte synaptic proximity and GAT-3 expression were quantified by isolating SYNJ1 or GAT-3 signal that co-registered with the rendered astrocyte (Supplementary Fig. S3) and normalized signal was reported as percent of astrocyte volume that contained co-registered signal. An average of ten astrocytes were imaged and quantified from both hemispheres across one to two slices per animal. For quantification of total SYNJ1 expression (Supplementary Fig. S4), *z*-stacks from each animal were cropped to exclude areas of abnormally low signal (i.e., due to the presence of large blood vessels or white matter) and SYNJ1 intensity was quantified and normalized to quadrant size. For determination of astrocyte association with D1- or D2-MSN terminals, digitally rendered astroglia were used to isolate co-registered mCherry, from labeled NAcore neurons, and co-registered SYNJ1 immunoreactivity (as shown in Fig. 2D). Next co-registration of isolated mCherry and SYNJ1 was determined using the Coloc module and quantified signal was normalized to volume of the rendered astrocyte. In all cases, determination of signal relative to noise was empirical and required exclusion of tissue surfaces (i.e., ~4 *z*-planes at the top and bottom of each stack) that exhibited higher signal intensity due to antibody accumulation and thus poor signal:noise.

### Vivo-morpholino knockdown

Bilateral cannulae (P1 Technologies) were placed 1.5 mm above the dlVP (+0.0 mm AP, ±2.2 mm ML, –5.9 mm DV) 5 days prior to the start of operant training and cannulae were kept patent by insertion of metal dummies (Component Supply Company, Inc.) of the same length. Immediately following sessions 7–9 of extinction training, rats received bilateral infusions (0.7 μL, 0.15 μL/min, 5 min diffusion) of an ezrin-targeted vivo-morpholino oligo (GCGCTCCGCAGGTTTCACTTCGTGA, 50 μM, Gene Tools, LLC), a GAT-3-targeted vivo-morpholino oligo (CCCAGTGCCTCCCTTAACACCTTGA, 50 μM, Gene Tools, LLC), or a standard control oligo with no expected binding target in the brain (50 μM, Gene Tools, LLC) under light isoflurane anesthesia (5% induction, 1.5% maintenance). Intracranial delivery of vivo-morpholino oligos for 3–5 consecutive days has been used previously to obtain effective knockdown of ezrin and other astroglial proteins 4–7 days after the last oligo infusion [25, 40–43].

### Locomotor testing

Locomotion was analyzed in a subset of animals that underwent morpholino-mediated GAT-3 or ezrin knockdown in the dlVP as described above. Five to seven days after morpholino delivery, rats were placed in a novel open field environment (42 × 42 × 32 cm) and photocell beam breaks (VersaMax) were used to quantify distance traveled during the 2-h session.

### Statistics

Data were analyzed using GraphPad Prism 9 (GraphPad Software) and are presented as scatter plots where possible, with cell and animal numbers clearly indicated. Statistical tests used for each analysis are stated in figure legends. Behavioral data are presented as mean ± SEM and were analyzed using one- or two-way ANOVA or Student’s *t*-test, unless unequal variance was observed between groups. Astroglial measures were first tested for normality using D’Agostino-Pearson omnibus normality test (Supplementary Table S1), followed by Kruskal–Wallis when one or more treatment groups were not normally distributed. Nested analyses are included for comparison (Supplementary Table S2). Non-Gaussian data are presented with bar at the median and full distribution shown in scatter. Effect size for each analysis is shown in Supplementary Table S3, where *η*^2^ < 0.06 indicates a small effect size, 0.06–0.14 indicates moderate effect size, and >0.14 indicates large effect size. Spearman’s correlation coefficient is listed for correlations and data are plotted as median ± 95% CI for each animal. For each test, *p* values < 0.05 were considered significant.

## Results

### Extinction of heroin self-administration upregulated GAT-3 expression in dlVP astrocytes

Astroglia in the dlVP of male and female rats were transduced with AAV5.gfaABC1D.Lck.GFP (Fig. 1A, B) and trained to self-administer heroin for 10 × 3 h daily sessions. Most rats then underwent 10 days of extinction training where heroin and cues were withheld and active lever pressing gradually decreased (Fig. 1D). One group of rats underwent 10 days of withdrawal in the home cage without extinction training (abstinent group). Forty-eight hours after the last extinction session, a group of rats underwent cued reinstatement for 15 min, where cues were delivered in response to active lever presses, but no heroin was infused (Fig. 1F). Astroglia were identified by their expression of membrane-GFP and their co-expression of immunolabeled GAT-3 was quantified and normalized to astrocyte volume (Supplementary Fig. S3).

We found increased GAT-3 expression after extinction of heroin self-administration that was not observed in rats withdrawn from heroin without extinction training (Fig. 1G, *N* = 45–98/3–8 cells/animals), indicating that GAT-3 upregulation resulted from extinction training. Levels of GAT-3 on dlVP astroglia correlated negatively with lever pressing during 15-min of cue-induced heroin seeking (Fig. 1H). Although cued heroin seeking did not alter GAT-3 expression when all dlVP astroglia were combined (Fig. 1G), when GAT-3 expression was analyzed at different stereological coordinates along the anterior-posterior axis [35], expression was selectively reduced in astroglia near +0.12 mm from bregma (Fig. 1I). This may indicate selective cue-induced GABA release at this rostrocaudal portion of the dlVP since GABA transporters may be removed from the surface after binding extracellular GABA [44].

### Synaptic proximity of astroglial processes was decreased during 15 min of reinstated heroin seeking

As a measure of morphological plasticity of astrocytes relative to synapses [25], we quantified the co-registration of labeled astroglia with the presynaptic marker SYNJ1 in the dlVP in rats that had extinguished heroin self-administration or in animals undergoing 15 min of cued reinstatement (Supplementary Fig. S3). Co-registration of immunolabeled SYNJ1 and the tagged astroglial membrane can be used as an index of proximity since the two markers will co-register when they are within the limit of resolution of confocal microscopy (i.e., ~200–250 nm) [25]. Although we found no changes in synaptic co-registration after heroin use overall (Fig. 1J), SYNJ1 co-registration correlated negatively with active lever pressing during cued reinstatement (Fig. 1K). When SYNJ1 co-registration was analyzed according to the position of astroglia along the anterior-posterior axis, synaptic retraction by astrocytes was localized to +0.12 mm from Bregma [35] (Fig. 1L), the same stereological coordinates where GAT-3 was reduced during cue exposure. Based on these findings, imaging for subsequent analyses was restricted to +0.12 ± 0.24 mm from Bregma to selectively sample from this subregion of the dlVP. Total levels of SYNJ1 were not changed by extinction or reinstatement compared to yoked controls (Supplementary Fig. S4) and neither SYNJ1 co-registration nor GAT-3 expression differed by sex (Supplementary Fig. S5).

### Astrocyte morphological plasticity in the dlVP was associated with D1-MSN terminals from the NAcore

Projections from NAcore D1- and D2-MSNs to the dlVP differentially regulate cued drug seeking and undergo unique measures of plasticity after withdrawal from cocaine self-administration [7]. To determine whether changes in SYNJ1 co-registration by dlVP astroglia during reinstated seeking were differentially associated with terminals from NAcore D1- or D2-MSNs, we trained D1- and D2-Cre rats to self-administer heroin (Fig. 1D) and quantified triple co-registration of labeled dlVP astroglia with immunolabeled SYNJ1 and mCherry-positive afferents labeled by virus delivery in the NAcore (Fig. 2A–D). We found increased co-registration of astrocytes with D1-MSN terminals after extinction of heroin self-administration, a measure that was reversed during 15-min cued reinstatement (Fig. 2E, *N* = 26–57/3–6 cells/animals) (Fig. 1L). In contrast, D2-MSN terminals had equivalent astroglial insulation across all three treatment conditions (Fig. 2E). These data indicate that changes in synaptic association of dlVP astroglia were selective for D1-MSN terminals.

### Adaptations in GAT-3 levels and astrocyte synaptic proximity during sucrose seeking differed from heroin seeking

**Fig. 3:**
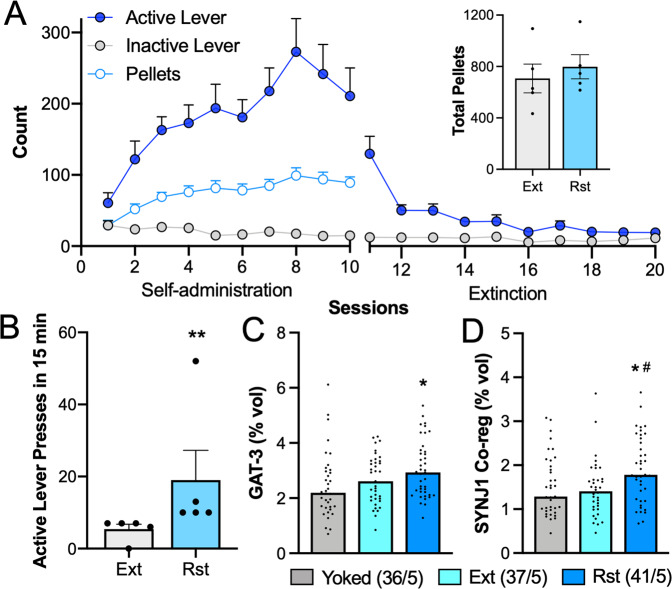
dlVP astroglia exhibited increased GAT-3 expression and synaptic co-registration during 15 min of cued sucrose seeking. **A** Male and female rats were trained to self-administer sucrose during daily 2 h sessions. After extinction training, rats reinstated sucrose seeking during 15-min exposure to sucrose-associated cues. Extinguished and reinstated rats self-administered similar amounts of sucrose over the course of self-administration (**A**, inset, Student’s *t*(8) = 0.6214, *p* = 0.5516). **B** Reinstated rats pressed more during 15 min of cue exposure than 15 min in the extinguished context during the last extinction session (Kolmogorov–Smirnov = 1.00, **p = 0.0079). **C** GAT-3 expression by dlVP astroglia was unchanged during extinction training but increased during cued reinstatement (Kruskal–Wallis = 7.699, *p* = 0.0213, *p = 0.0168 vs. Yoked using Dunn’s post hoc test). **D** SYNJ1 co-registration by dlVP astroglia was also elevated during cued reinstatement of sucrose seeking (Kruskal–Wallis = 9.323, *p* = 0.0095, ^#^*p* = 0.0176 vs. Ext using Dunn’s post hoc test). Data shown as mean ± SEM (**A**, **B**) or median (**C**, **D**). *N* is shown in legend as cells/animals (**C**, **D**). Yoked yoked cues, Ext extinguished, Rst 15-min reinstated.

To determine whether the reductions in GAT-3 expression and astrocyte synaptic proximity observed during cued heroin seeking in the rostral dlVP occurred during natural reward seeking, separate rats were trained to self-administer sucrose (Fig. 3A, B). We found that although extinction training had no effect on GAT-3 expression (Fig. 3C), both GAT-3 levels and SYNJ1 co-registration were increased during 15 min of cued sucrose seeking (Fig. 3C, D, *N* = 36–41/5 cells/animals).

### GAT-3 upregulation and synaptic association of dlVP astrocytes were necessary for extinction of heroin seeking

**Fig. 4:**
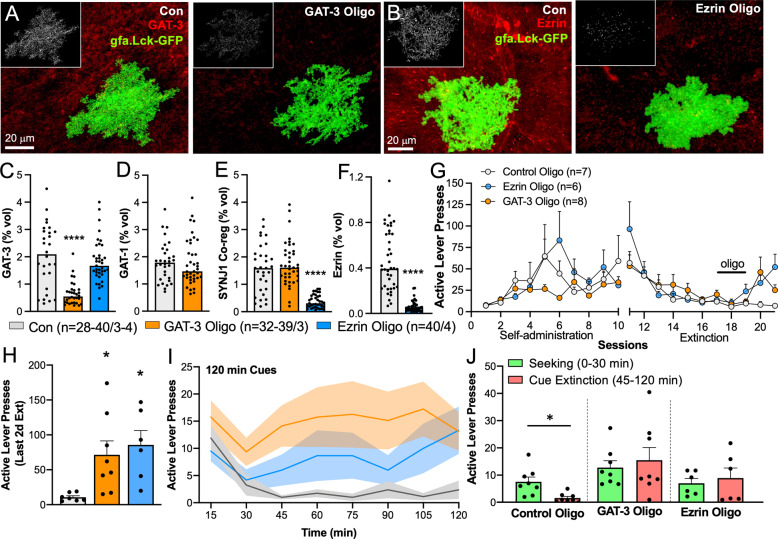
GAT-3 expression and synaptic insulation by dlVP astroglia suppressed heroin seeking during extinction and reinstatement. Vivo-morpholino oligomers targeted to GAT-3 (**A**) or ezrin (**B**) were tested for their ability to reduce GAT-3 overexpression and synaptic insulation by dlVP astroglia. Delivery of the GAT-3 oligo reduced GAT-3 expression (**C**, Kruskal–Wallis = 39.32, *p* < 0.0001, ****p < 0.0001 Con vs. GAT-3 Oligo using Dunn’s test), but did not impact expression of GAT-1 (**D**, Kolmogorov–Smirnov = 0.2147, *p* = 0.3923) or synaptic proximity of the astroglial membrane (**E**, Kruskal–Wallis = 56.60, *p* < 0.0001, *p* = 0.8409 Con vs. GAT-3 Oligo using Dunn’s test). Delivery of the ezrin oligo reduced ezrin expression (**F**, Kolmogorov–Smirnov = 0.8750, ****p < 0.0001) and SYNJ1 co-registration (**E**, ****p < 0.0001 Con vs. Ezrin Oligo using Dunn’s test) compared to a control oligo, but did not impact GAT-3 levels (**C**, *p* > 0.9999 Con vs. Ezrin Oligo using Dunn’s test). **G** Animals were trained to self-administer heroin and were extinguished for 6 days before receiving dlVP infusions of GAT-3, ezrin, or control oligo for 3 consecutive days via intracranial cannulae. Active lever pressing was increased during the final two extinction sessions in animals that received either the GAT-3 or ezrin oligo compared to animals that received a control oligo (**H**, Welch’s ANOVA *W*(2,7.95) = 10.27, *p* = 0.0063, *p = 0.0353 GAT-3 Oligo vs. Control Oligo; *p = 0.0278 Ezrin Oligo vs. Control Oligo using Dunnett’s test). **I** During 120 min of cued reinstatement, control oligo-treated rats underwent within-session cue extinction after 45 min (two-way ANOVA treatment *F*(2,19) = 4.321, *p* = 0.0284, Control Oligo 15 vs. 45 min, *p* = 0.0067 using Dunnett’s test). Animals that underwent GAT-3 (15 vs. 45 min, *p* = 0.9101 using Dunnett’s test) or ezrin knockdown (15 vs. 45 min, *p* = 0.7568 using Dunnett’s test) did not extinguish their cued lever pressing during the 2-h session. **J** Control oligo-treated rats reinstated seeking behavior during 30 min of cue exposure (Seek), after which unrewarded seeking was extinguished (45–120 min, cue extinction; *t*(7) = 4.421, *p = 0.0093 using a planned comparisons *t*-test with a Bonferroni adjustment). Animals that underwent GAT-3 (*t*(7) = 0.7105, *p* > 0.999) or ezrin knockdown (*t*(5) = 0.9810, *p* > 0.999) did not extinguish cued seeking over the course of the session. Data shown as median (**C**–**F**) or mean ± SEM (**G**–**J**). *N* shown in legend as cells/animals (**C–F**). Con control oligo.

Interestingly, the astroglial measurements of GAT-3 expression and SYNJ1 co-registration in rats undergoing cued reinstatement correlated negatively with active lever pressing (Fig. 1H, K), suggesting a protective effect of these adaptations on relapse behavior. To evaluate this possibility, we used a vivo-morpholino oligomer strategy to knockdown GAT-3 or ezrin, an actin-binding protein needed to form perisynaptic astroglial processes [25, 45]. This strategy allows for relatively rapid, robust, and reversible knockdown of target proteins in a region of interest, but does not necessitate the use of transgenic animals or extended time periods for viral incubation [40]. Delivery of the GAT-3 oligo reduced GAT-3 expression compared with a control oligo delivered contralaterally in untreated animals (Fig. 4A, C and Supplementary Fig. S1, *N* = 28–40/3–4 cells/animals). The GAT-3 oligo had no effect on GAT-1 expression, a GABA transporter expressed perisynaptically by both neurons and astroglia [30] (Fig. 4D), or on SYNJ1 co-registration by astrocytes (Fig. 4E). Similarly, the ezrin-targeted oligo produced robust knockdown of ezrin expression (Fig. 4B, F) and a corresponding reduction in SYNJ1 co-registration by astrocytes (Fig. 4E), but did not affect GAT-3 expression (Fig. 4C).

Rats were trained to self-administer heroin and received bilateral oligo infusions in the dlVP immediately following sessions 7–9 of extinction training (*N* = 6–8/grp). Using this strategy, we compared active lever pressing in three treatment groups, rats that were infused bilaterally with a control oligomer and those that underwent either bilateral GAT-3 or ezrin knockdown. Compared to controls, knockdown of either GAT-3 or ezrin appeared to reverse extinction training and elevated heroin seeking in the extinguished context (Fig. 4G, H). During 120 min of cued reinstatement, neither GAT-3 nor ezrin oligos markedly altered the initial seeking response to cues, defined as occurring during the first 30 min of the reinstatement session where control rats show peak active lever pressing [25, 46], but prevented within-trial extinction of seeking, defined as 45–120 min after initiating the reinstatement test, where control rats show declining active lever pressing often referred to as within-trial extinction [25] (i.e., cue extinction, Fig. 4I). While control rats showed significant extinction during 2 h of cue exposure, neither the GAT-3 nor ezrin knockdown groups underwent within-trial cue extinction (Fig. 4J). Remarkably, the same knockdown strategy had no impact on measures of seeking in sucrose-trained animals (Supplementary Fig. S6). Importantly, GAT-3 or ezrin knockdown did not impact lever discrimination during the final extinction session or during cued reinstatement and had no impact on general locomotion in a novel open field (Supplementary Fig. S7, *N* = 6–7/grp), illustrating that the increase in lever pressing in oligo-treated animals reflected motivation to acquire heroin and not nonspecific increases in lever pressing or motor activity. Together, these data show that increased GAT-3 expression and synaptic insulation by dlVP astroglia during extinction training suppress heroin seeking and are necessary for extinction of responding.

## Discussion

Excitatory transmission onto D1- and D2-MSNs in the NAcore undergoes marked plasticity after drug use that regulates drug seeking in animal models of relapse and NAcore perisynaptic astroglia critically regulate glutamatergic transmission and cue-induced drug seeking [25]. The dlVP contains a dense and behaviorally critical accumbens axon terminal field that regulates drug seeking in animal models, yet nothing is known regarding how astroglia in the dlVP contribute to drug relapse. We examined the proximity of astrocytes to dlVP synapses and levels of the astroglial GABA transporter GAT-3 in a rat model of heroin use and relapse. We show that heroin self-administration combined with extinction training, but not withdrawal without extinction training, upregulated GAT-3 in dlVP astroglia and that astrocyte proximity to D1-MSN, but not D2-MSN synapses was also increased. When either of these adaptations was prevented by directly knocking down GAT-3 or indirectly reducing astrocyte synaptic proximity by knocking down ezrin, rats lost the ability to extinguish drug seeking. This was revealed when rats were placed into the extinguished context after knockdown without heroin and showed marked increases in active lever pressing. Moreover, when these rats underwent cued reinstatement, there was a clear inability to extinguish cued responding over the course of the trial. Regulation of extinction responding by these astroglial adaptations occurred selectively after heroin use, since neither GAT-3 or ezrin knockdown impacted extinguished or reinstated lever pressing in sucrose-trained rats. Together these findings suggest that astrocyte adaptations necessary for extinguished responding are selective for heroin self-administration.

### Impact of heroin-induced changes in GAT-3 and astrocyte synaptic proximity on GABAergic transmission

GAT-3 is a primary regulator of extracellular GABA concentrations [22]. Through high affinity GABA uptake, GAT-3 can shape GABAergic synaptic transmission by directly lowering synaptic GABA when localized perisynaptically and by reducing GABA spillover to extrasynaptic GABA receptors and adjacent GABAergic synapses. Although electron microscopy reveals that GAT-3 is largely localized outside of perisynaptic processes [30], the increase in GAT-3 produced by heroin extinction training would compete with GABA receptors for extracellular GABA. The increase in astrocyte proximity to D1-MSN, but not D2-MSN synapses in the dlVP after extinction training would be expected to have a similar overall effect, both by placing surface GAT-3 closer to the synaptic cleft and functioning as a steric inhibitor of synaptic GABA spillover, decreasing diffusion of GABA into the extrasynaptic space, as occurs with glutamatergic synapses [24]. Indeed, the reduction in GAT-3 after 15 min of cued reinstatement may reflect increased GABA binding and consequently increased trafficking of GAT-3 off the astroglial surface, as occurs for GAT-1 [44], or activation of signaling cascades linked to GABA transporter internalization [47].

### Astrocyte interaction with the dlVP circuitry and extinction of drug seeking

Neuronal activity in the VP is necessary to coordinate adaptive behavioral responses to motivationally salient information, including cues that reinstate drug seeking [48–50]. The VP has been divided into dorsolateral and ventromedial portions based on inputs from the NAcore and shell, respectively [36] and notable subdivisions have been observed within the structure. For example, neurons in the rostral VP receive more glutamatergic input and exhibit electrophysiological properties similar to striatal MSNs, while caudal portions of the VP receive denser GABAergic input [38]. Notably, the rostral VP has been linked with cued reinstatement of seeking, since its inhibition prevents cued responding for cocaine in extinguished rats [50]. These findings are consistent with our observation that 15 min of cued reinstatement elicited astroglial plasticity selectively near +0.12 mm from bregma, coordinates consistent with a rostral designation in the previous study. Instead, caudal portions of the VP are linked to increased reward consumption and hedonic liking, mediated by μ-opioid receptors [51].

Since activation of neurons in the rostral VP is necessary for cued reinstatement of cocaine seeking [50], it is perhaps surprising that GABAergic projections from the NAcore to the dlVP are also necessary to reinstate seeking [52]. This apparent paradox may relate to the intrinsic neural circuitry within the dlVP. The dlVP consists of two primary neuronal subtypes, GABAergic (~70–75%) and glutamatergic (~20–25%) [16, 53], and recent studies indicate that activation of GABA neurons produces drug seeking while activation of glutamatergic cells inhibits drug seeking and elicits anhedonia [8, 15, 16]. Though both cell types receive GABAergic input from D1- and D2-MSNs, a recent retrograde tracing study reveals that D1-MSN input is more prominent onto glutamatergic neurons [16]. Also, cocaine self-administration or repeated cocaine injections produce presynaptic inhibition of D2 afferents and potentiate D1-MSN inputs [7, 8]. Reversal of D1-MSN potentiation prevents sensitized locomotor responding, a surrogate measure of increased motivation such as occurs in cued seeking [8]. Thus, activity of D1-MSN inputs appears to promote motivated behaviors while activity of D2-MSN afferents inhibits motivated behaviors, indicating that astroglial plasticity surrounding D1-MSN terminals could be highly consequential in regulating drug seeking by promoting extinction of seeking. The present findings support this possibility as follows: (1) the dense D1-MSN input onto glutamatergic neurons and extinction-induced astrocyte association with D1-MSN afferents and overall elevation of GAT-3 are consistent with an astrocyte-dependent reduction in GABAergic inhibition of glutamatergic neurons to promote extinction. (2) Reversal of extinction-induced astrocyte association with D1-MSN synapses and GAT-3 elevation after 15 min of cued reinstatement would promote inhibition of glutamatergic neurons and support cue-induced reinstatement. Future studies on the impact of upregulated astroglial GAT-3 on the physiology of post-synaptic GABAergic and glutamatergic neurons after extinction of heroin self-administration are needed to validate these hypotheses. Future studies on the effect of GAT-3 or ezrin knockdown on heroin seeking after home cage abstinence are also warranted to further support our hypothesis that astroglial adaptations are necessary for extinction of heroin seeking.

Although the NAshell, which projects to the ventromedial VP, is often cited as a critical nucleus for extinction learning [54], our findings argue that extinction is also mediated by astrocytes in the dlVP through action on D1-MSN projections from the NAcore. These data are consistent with a growing literature that points to plasticity in the NAcore as well as the NAshell as an important factor in extinction learning, including observations that active refraining from drug seeking promotes potentiation of NAcore D2-MSNs [14, 55].

### Comparison with previous findings in the NAcore

In our previous studies, we noted robust and transient astroglial plasticity in the NAcore associated with glutamatergic transmission during relapse [25]. In the present study, we confirm that astrocytes also exhibit morphological plasticity in response to GABAergic transmission. Interestingly, in the NAcore cue-induced glutamatergic transmission triggers transient increases in synaptic insulation by astrocyte processes, whereas GABAergic transmission in the dlVP produced the opposite effect, resulting in retraction of astrocyte processes from synapses. Although the VP receives excitatory input from the subthalamus [56, 57], ventral tegmental area [58], paraventricular nucleus of the thalamus [59], amygdala [60, 61], and prefrontal cortex [62] as well as local glutamatergic neurons [63], little is known about the contribution of glutamatergic signaling in the VP to drug seeking or extinction of seeking and this remains an important direction for future investigations.

### Effect of sex on astroglial plasticity relevant to heroin seeking

Research by others has uncovered sex differences in synaptic adjacency of astrocytes in the NAcore following cocaine self-administration and extinction [64]. In the present study, we found no effect of sex on the basic of immunohistochemical or morphological measures analyzed. Instead, we found that GAT-3 was upregulated during extinction in both male and female rats. As noted in previous publications, astroglial measures were not normally distributed across the population of cells analyzed (Supplementary Table S1) and a similar degree of variability was noted in data gathered from male and female subjects (see Supplementary Fig. S5). Since we did not assess the estrous stage of female rats during behavioral testing or tissue collection, the possibility remains that differences in cycling hormones may have some impact on the measures of interest presented here.

### Natural rewards and dlVP astroglial adaptations

Consistent with the possible role for changes in astroglial GAT-3 and synaptic proximity selectively impacting extinction of drug seeking, sucrose self-administration followed by extinction training did not alter either measure. However, after 15 min of cue-reinstated sucrose seeking, both astroglial measurements were elevated, opposite to what occurred during heroin reinstatement, and would be expected to promote within-trial extinction of cue-induced sucrose seeking relative to heroin seeking. Indeed, we found that within-trial extinction during cued sucrose seeking occurred more rapidly than during heroin seeking (Supplementary Fig. S8), consistent with this interpretation. Remarkably, when either GAT-3 or ezrin were knocked down during extinction after sucrose self-administration, we found no effect on active lever pressing in the extinguished context or in the presence of sucrose-associated cues. These findings indicate that astroglial adaptations are necessary following heroin use to permit extinction of seeking.

### Astrocyte-mediated extinction of heroin seeking: relevance for addiction-related behaviors

In our animal model, heroin seeking behavior was extinguished after self-administration by removal of the reinforcer, a process that engaged plasticity in dlVP astroglia. In humans recovering from substance use disorder, cues and contexts previously associated with drug use can elicit strong craving and stimulate relapse [65–67] and the ability to override conditioned responses poses a potential therapeutic strategy to reduce drug use in patients with substance use disorder [68]. Extinction of conditioned drug-seeking responses involves plasticity in cortical and subcortical brain regions (reviewed in [69]), and was recently shown to engage glutamatergic neurons within the VP [16]. Our findings expand upon these discoveries, demonstrating an important role for dlVP astroglia in permitting extinction of drug-seeking responses through selective engagement with D1-MSN afferents from the NAcore. Manipulating extinction of drug seeking remains elusive in human patients due to pervasiveness of cue-drug reward associations [70]. However, modern extinction-based approaches for reducing drug-seeking and relapse have shown modest promise in clinically reducing drug use [71–75] and understanding mechanisms underlying extinction is a rational approach to improving its efficacy clinically. Thus, the finding that astrocyte adaptations can promote extinction of seeking in the absence of reward serves as a promising avenue for reducing cue reactivity in human patients. For example, the recent generation of a GAT-3 overexpression vector [76] might serve as a useful tool for investigating this hypothesis.

## Conclusions and perspectives

Opioid use disorder has reached epidemic proportions in the USA. We define critical astroglial adaptations in the dlVP that endogenously oppose drug-seeking behavior during withdrawal by supporting refraining from heroin seeking after extinction training. These adaptations are reversed during presentation of heroin cues, which contributes to more intense seeking in response to heroin cues compared with cues predicting a natural reward. These findings, combined with our previous studies in the NAcore, highlight that astroglia are uniquely impacted by withdrawal from drug use compared with natural rewards and that their morphological plasticity and expression of transporters are highly plastic during relapse in a manner that modulates drug seeking [25, 27]. This work underscores the importance of understanding the contribution of astroglial dynamics to neural signaling and opens new molecular targets (e.g., GAT-3 or ezrin) for developing therapeutic interventions in treating opioid use disorder.

## Supplementary information

Supplementary Figures and Legends

## References

[1] Chalhoub RM, Kalivas PW. Non-opioid treatments for opioid use disorder: rationales and data to date. Drugs. 2020;80:1509–24. 10.1007/s40265-020-01373-1. 10.1007/s40265-020-01373-1 PMC7541583 32776315

[2] Koob GF (2020). Neurobiology of opioid addiction: opponent process, hyperkatifeia, and negative reinforcement. Biol Psychiatry.

[3] Koob GF, Volkow ND (2016). Neurobiology of addiction: a neurocircuitry analysis. Lancet Psychiatry.

[4] Pardo-Garcia TR, Garcia-Keller C, Penaloza T, Richie CT, Pickel J, Hope BT (2019). Ventral pallidum is the primary target for accumbens D1 projections driving cocaine seeking. J Neurosci.

[5] Nall RW, Heinsbroek JA, Nentwig TB, Kalivas PW, Bobadilla AC. Circuit selectivity in drug versus natural reward seeking behaviors. J Neurochem. 2021;157:1450–72. 10.1111/jnc.15297. 10.1111/jnc.15297 PMC8178159 33420731

[6] Kalivas PW (2009). The glutamate homeostasis hypothesis of addiction. Nat Rev Neurosci.

[7] Heinsbroek JA, Neuhofer DN, Griffin WC, Siegel GS, Bobadilla AC, Kupchik YM (2017). Loss of plasticity in the D2-accumbens pallidal pathway promotes cocaine seeking. J Neurosci.

[8] Creed M, Ntamati NR, Chandra R, Lobo MK, Luscher C (2016). Convergence of reinforcing and anhedonic cocaine effects in the ventral pallidum. Neuron.

[9] Pascoli V, Terrier J, Espallergues J, Valjent E, O’Connor EC, Luscher C (2014). Contrasting forms of cocaine-evoked plasticity control components of relapse. Nature.

[10] Moussawi K, Pacchioni A, Moran M, Olive MF, Gass JT, Lavin A (2009). N-Acetylcysteine reverses cocaine-induced metaplasticity. Nat Neurosci.

[11] Hearing M, Graziane N, Dong Y, Thomas MJ (2018). Opioid and psychostimulant plasticity: targeting overlap in nucleus accumbens glutamate signaling. Trends Pharm Sci.

[12] Smith RJ, Lobo MK, Spencer S, Kalivas PW (2013). Cocaine-induced adaptations in D1 and D2 accumbens projection neurons (a dichotomy not necessarily synonymous with direct and indirect pathways). Curr Opin Neurobiol.

[13] Calipari ES, Bagot RC, Purushothaman I, Davidson TJ, Pena CJ, Walker DM (2016). In vivo imaging identifies temporal signature of D1 and D2 medium spiny neurons in cocaine reward. Proc Natl Acad Sci USA.

[14] Roberts-Wolfe D, Bobadilla AC, Heinsbroek JA, Neuhofer D, Kalivas PW (2018). Drug refraining and seeking potentiate synapses on distinct populations of accumbens medium spiny neurons. J Neurosci.

[15] Tooley J, Marconi L, Alipio JB, Matikainen-Ankney B, Georgiou P, Kravitz AV, et al. Glutamatergic ventral pallidal neurons modulate activity of the habenula-tegmental circuitry and constrain reward seeking. Biol Psychiatry. 2018;83:1012–23. 10.1016/j.biopsych.2018.01.003. 10.1016/j.biopsych.2018.01.003 PMC5972062 29452828

[16] Heinsbroek JA, Bobadilla AC, Dereschewitz E, Assali A, Chalhoub RM, Cowan CW (2020). Opposing regulation of cocaine seeking by glutamate and GABA neurons in the ventral pallidum. Cell Rep..

[17] Wang J, Li KL, Shukla A, Beroun A, Ishikawa M, Huang X (2021). Cocaine triggers astrocyte-mediated synaptogenesis. Biol Psychiatry.

[18] Ehlers MD (2005). Synapse formation: astrocytes spout off. Curr Biol.

[19] Eroglu C, Allen NJ, Susman MW, O’Rourke NA, Park CY, Ozkan E (2009). Gabapentin receptor alpha2delta-1 is a neuronal thrombospondin receptor responsible for excitatory CNS synaptogenesis. Cell.

[20] Murphy-Royal C, Dupuis JP, Varela JA, Panatier A, Pinson B, Baufreton J (2015). Surface diffusion of astrocytic glutamate transporters shapes synaptic transmission. Nat Neurosci.

[21] Danbolt NC (2001). Glutamate uptake. Prog Neurobiol.

[22] Kersante F, Rowley SC, Pavlov I, Gutierrez-Mecinas M, Semyanov A, Reul JM (2013). A functional role for both -aminobutyric acid (GABA) transporter-1 and GABA transporter-3 in the modulation of extracellular GABA and GABAergic tonic conductances in the rat hippocampus. J Physiol.

[23] Perez-Alvarez A, Navarrete M, Covelo A, Martin ED, Araque A (2014). Structural and functional plasticity of astrocyte processes and dendritic spine interactions. J Neurosci.

[24] Henneberger C, Bard L, Panatier A, Reynolds JP, Kopach O, Medvedev NI (2020). LTP induction boosts glutamate spillover by driving withdrawal of perisynaptic astroglia. Neuron.

[25] Kruyer A, Scofield MD, Wood D, Reissner KJ, Kalivas PW (2019). Heroin cue-evoked astrocytic structural plasticity at nucleus accumbens synapses inhibits heroin seeking. Biol Psychiatry.

[26] Scofield MD, Kalivas PW. Astrocytic dysfunction and addiction: consequences of impaired glutamate homeostasis. Neuroscientist. 2014;20:610–22. 10.1177/1073858413520347. 10.1177/1073858413520347 PMC4913887 24496610

[27] Kruyer A, Kalivas PW (2020). Astrocytes as cellular mediators of cue reactivity in addiction. Curr Opin Pharm.

[28] Zahm DS (1989). The ventral striatopallidal parts of the basal ganglia in the rat. II. Compartmentation of ventral pallidal efferents. Neuroscience.

[29] Masson J, Sagne C, Hamon M, El Mestikawy S (1999). Neurotransmitter transporters in the central nervous system. Pharm Rev.

[30] Melone M, Ciappelloni S, Conti F (2015). A quantitative analysis of cellular and synaptic localization of GAT-1 and GAT-3 in rat neocortex. Brain Struct Funct.

[31] Minelli A, DeBiasi S, Brecha NC, Zuccarello LV, Conti F (1996). GAT-3, a high-affinity GABA plasma membrane transporter, is localized to astrocytic processes, and it is not confined to the vicinity of GABAergic synapses in the cerebral cortex. J Neurosci.

[32] Scofield MD, Li H, Siemsen BM, Healey KL, Tran PK, Woronoff N (2016). Cocaine self-administration and extinction leads to reduced glial fibrillary acidic protein expression and morphometric features of astrocytes in the nucleus accumbens core. Biol Psychiatry.

[33] McPherson PS, Garcia EP, Slepnev VI, David C, Zhang X, Grabs D (1996). A presynaptic inositol-5-phosphatase. Nature.

[34] Sunkin SM, Ng L, Lau C, Dolbeare T, Gilbert TL, Thompson CL (2013). Allen Brain Atlas: an integrated spatio-temporal portal for exploring the central nervous system. Nucleic Acids Res.

[35] Paxinos G, Watson C. The rat brain in stereotaxic coordinates. 7th ed. London, UK: Academic Press; 2013.

[36] Heimer L, Zahm DS, Churchill L, Kalivas PW, Wohltmann C (1991). Specificity in the projection patterns of accumbal core and shell in the rat. Neuroscience.

[37] Klitenick MA, Deutch AY, Churchill L, Kalivas PW (1992). Topography and functional role of dopaminergic projections from the ventral mesencephalic tegmentum to the ventral pallidum. Neuroscience.

[38] Kupchik YM, Kalivas PW (2013). The rostral subcommissural ventral pallidum is a mix of ventral pallidal neurons and neurons from adjacent areas: an electrophysiological study. Brain Struct Funct.

[39] Farrell MR, Ruiz CM, Castillo E, Faget L, Khanbijan C, Liu S (2019). Ventral pallidum is essential for cocaine relapse after voluntary abstinence in rats. Neuropsychopharmacology.

[40] Reissner KJ, Sartor GC, Vazey EM, Dunn TE, Aston-Jones G, Kalivas PW (2012). Use of vivo-morpholinos for control of protein expression in the adult rat brain. J Neurosci Methods.

[41] Reissner KJ, Gipson CD, Tran PK, Knackstedt LA, Scofield MD, Kalivas PW (2015). Glutamate transporter GLT-1 mediates N-acetylcysteine inhibition of cocaine reinstatement. Addict Biol.

[42] Shen HW, Scofield MD, Boger H, Hensley M, Kalivas PW (2014). Synaptic glutamate spillover due to impaired glutamate uptake mediates heroin relapse. J Neurosci.

[43] Garcia-Keller C, Neuhofer D, Bobadilla AC, Spencer S, Chioma VC, Monforton C (2019). Extracellular matrix signaling through beta3 integrin mediates cocaine cue-induced transient synaptic plasticity and relapse. Biol Psychiatry.

[44] Quick MW (2002). Substrates regulate gamma-aminobutyric acid transporters in a syntaxin 1A-dependent manner. Proc Natl Acad Sci USA.

[45] Derouiche A, Frotscher M (2001). Peripheral astrocyte processes: monitoring by selective immunostaining for the actin-binding ERM proteins. Glia.

[46] Gipson CD, Kupchik YM, Shen H, Reissner KJ, Thomas CA, Kalivas PW (2013). Relapse induced by cues predicting cocaine depends on rapid, transient synaptic potentiation. Neuron.

[47] Scimemi A (2014). Structure, function, and plasticity of GABA transporters. Front Cell Neurosci.

[48] McFarland K, Kalivas PW (2001). The circuitry mediating cocaine-induced reinstatement of drug-seeking behavior. J Neurosci.

[49] Smith KS, Tindell AJ, Aldridge JW, Berridge KC (2009). Ventral pallidum roles in reward and motivation. Behav Brain Res.

[50] Mahler SV, Vazey EM, Beckley JT, Keistler CR, McGlinchey EM, Kaufling J (2014). Designer receptors show role for ventral pallidum input to ventral tegmental area in cocaine seeking. Nat Neurosci.

[51] Smith KS, Berridge KC (2005). The ventral pallidum and hedonic reward: neurochemical maps of sucrose “liking” and food intake. J Neurosci.

[52] Stefanik MT, Kupchik YM, Brown RM, Kalivas PW (2013). Optogenetic evidence that pallidal projections, not nigral projections, from the nucleus accumbens core are necessary for reinstating cocaine seeking. J Neurosci.

[53] Faget L, Zell V, Souter E, McPherson A, Ressler R, Gutierrez-Reed N (2018). Opponent control of behavioral reinforcement by inhibitory and excitatory projections from the ventral pallidum. Nat Commun.

[54] Gibson GD, Millan EZ, McNally GP (2019). The nucleus accumbens shell in reinstatement and extinction of drug seeking. Eur J Neurosci.

[55] Chioma VC, Kruyer A, Bobadilla AC, Angelis A, Ellison Z, Hodebourg R, et al. Heroin seeking and extinction from seeking activate matrix metalloproteinases at synapses on distinct subpopulations of accumbens cells. Biol Psychiatry. 2021;89:947–58. 10.1016/j.biopsych.2020.12.004. 10.1016/j.biopsych.2020.12.004 PMC8434769 33579535

[56] Groenewegen HJ, Berendse HW (1990). Connections of the subthalamic nucleus with ventral striatopallidal parts of the basal ganglia in the rat. J Comp Neurol.

[57] Turner MS, Lavin A, Grace AA, Napier TC (2001). Regulation of limbic information outflow by the subthalamic nucleus: excitatory amino acid projections to the ventral pallidum. J Neurosci.

[58] Hnasko TS, Hjelmstad GO, Fields HL, Edwards RH (2012). Ventral tegmental area glutamate neurons: electrophysiological properties and projections. J Neurosci.

[59] Perry CJ, McNally GP (2013). A role for the ventral pallidum in context-induced and primed reinstatement of alcohol seeking. Eur J Neurosci.

[60] Mitrovic I, Napier TC (1998). Substance P attenuates and DAMGO potentiates amygdala glutamatergic neurotransmission within the ventral pallidum. Brain Res.

[61] Maslowski-Cobuzzi RJ, Napier TC (1994). Activation of dopaminergic neurons modulates ventral pallidal responses evoked by amygdala stimulation. Neuroscience.

[62] Vertes RP (2004). Differential projections of the infralimbic and prelimbic cortex in the rat. Synapse.

[63] Levi LA, Inbar K, Nachshon N, Bernat N, Gatterer A, Inbar D (2020). Projection-specific potentiation of ventral pallidal glutamatergic outputs after abstinence from cocaine. J Neurosci.

[64] Brown N. Sex differences in addiction: the effects of cocaine self-administration and abstinence on astrocytes in the nucleus accumbens of female rats. 2019. 10.17615/9mxp-4f81.

[65] Garland EL, Bryan CJ, Kreighbaum L, Nakamura Y, Howard MO, Froeliger B (2018). Prescription opioid misusing chronic pain patients exhibit dysregulated context-dependent associations: Investigating associative learning in addiction with the cue-primed reactivity task. Drug Alcohol Depend.

[66] Benvegnù G, Tommasi F, Ferraro S, Libener E, Di Chio M, Bosi S, et al. Smokers “context reactivity” in virtual domestic environments. Eur Addict Res. 2021:1–8. 10.1159/000515301. [Epub ahead of print]. 10.1159/000515301 33940577

[67] Saraiya TC, Jarnecke AM, Jones J, Brown DG, Brady KT, Back SE (2021). Laboratory-induced stress and craving predict opioid use during follow-up among individuals with prescription opioid use disorder. Drug Alcohol Depend.

[68] O’Brien CP, Childress AR, McLellan AT, Ehrman R (1993). Developing treatments that address classical conditioning. NIDA Res Monogr.

[69] Gass JT, Chandler LJ (2013). The plasticity of extinction: contribution of the prefrontal cortex in treating addiction through inhibitory learning. Front Psychiatry.

[70] Marissen MA, Franken IH, Blanken P, van den Brink W, Hendriks VM (2007). Cue exposure therapy for the treatment of opiate addiction: results of a randomized controlled clinical trial. Psychother Psychosom.

[71] Powell J, Gray J, Bradley B (1993). Subjective craving for opiates: evaluation of a cue exposure protocol for use with detoxified opiate addicts. Br J Clin Psychol.

[72] Lee J, Lim Y, Graham SJ, Kim G, Wiederhold BK, Wiederhold MD (2004). Nicotine craving and cue exposure therapy by using virtual environments. Cyberpsychol Behav.

[73] Pericot-Valverde I, Garcia-Rodriguez O, Gutierrez-Maldonado J, Secades-Villa R (2015). Individual variables related to craving reduction in cue exposure treatment. Addict Behav.

[74] Du J, Fan C, Jiang H, Sun H, Li X, Zhao M (2014). Biofeedback combined with cue-exposure as a treatment for heroin addicts. Physiol Behav.

[75] Germeroth LJ, Carpenter MJ, Baker NL, Froeliger B, LaRowe SD, Saladin ME (2017). Effect of a brief memory updating intervention on smoking behavior: a randomized clinical trial. JAMA Psychiatry.

[76] Yu X, Taylor AMW, Nagai J, Golshani P, Evans CJ, Coppola G (2018). Reducing astrocyte calcium signaling in vivo alters striatal microcircuits and causes repetitive behavior. Neuron.

