# Supplementary material for: Astrocytes in the ventral pallidum extinguish heroin seeking through GAT-3 upregulation and morphological plasticity at D1-MSN terminals
Source: Mol Psychiatry. 2021 Oct 12;27(2):855–64. doi: 10.1038/s41380-021-01333-5 (PMC9054673; doi:10.1038/s41380-021-01333-5)
Supplement: Supplementary file 1 — Supplementary Figures and Legends [file 41380_2021_1333_MOESM1_ESM.docx]

**Supplementary Figures and Legends**


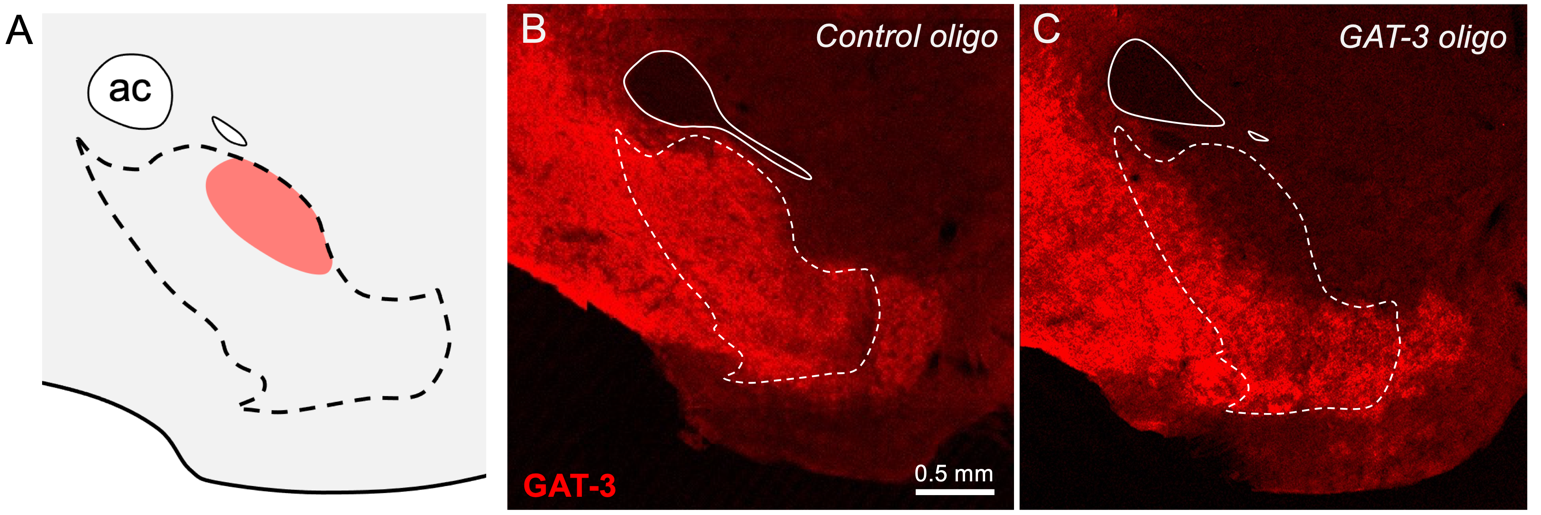


**Figure S1. Region of interest and representative GAT-3 knockdown.** (**A**) Schematic of VP (dashed line) with dlVP indicated in red. Solid line shows anterior commissure (ac). To test for efficacy of the GAT-3 oligo, a group of animals received unilateral infusions of control (**B**) or GAT-3 oligo (**C**) for 3 days via intracranial cannulae. GAT-3 immunolabeling (red, **B**-**C**) revealed that the GAT-3 oligo produced GAT-3 knockdown selectively in the dlVP compared with the contralateral hemisphere.


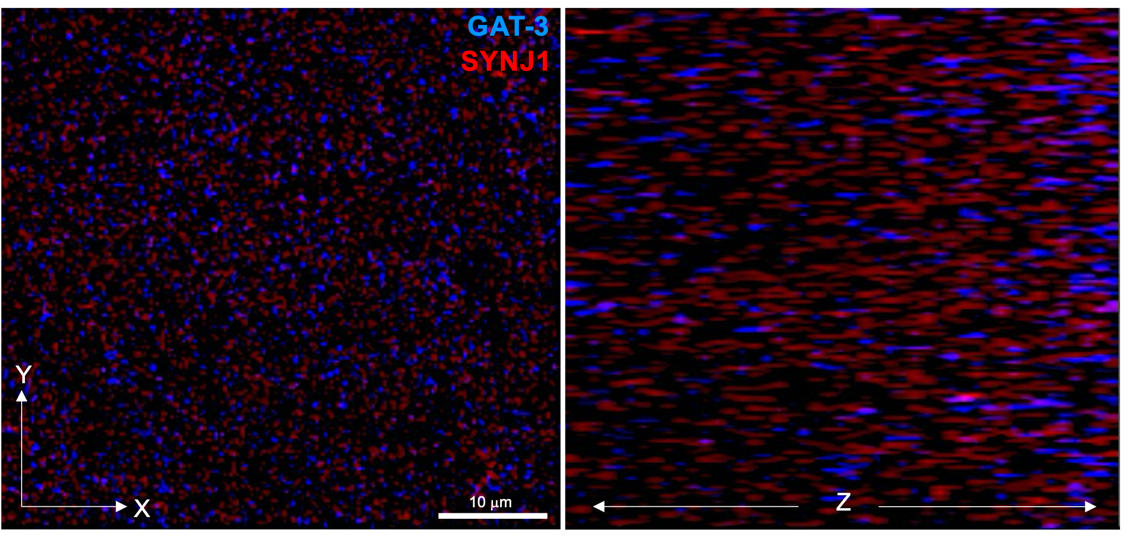


**Figure S2. Antibody penetration in 50-μm tissue slices.** GAT-3 (blue) and SYNJ1 immunoreactivity (red) was evenly distributed throughout confocal stacks analyzed for co-registration, reflecting antibody penetration in 50-μm tissue.


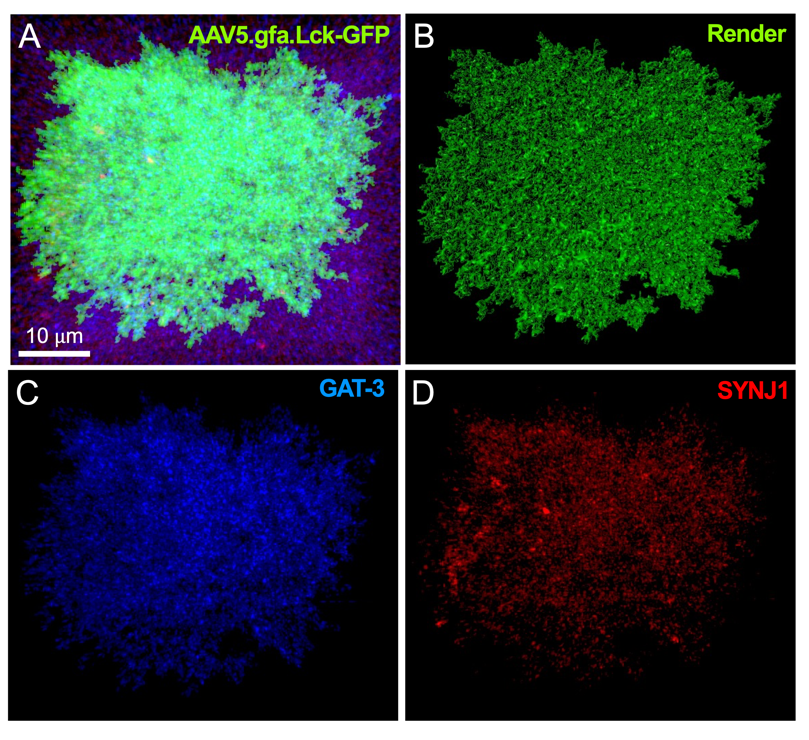


**Figure S3. Astroglial GAT-3 and co-registration with SYNJ1.** (**A**) Astroglia in the dlVP were labeled with membrane-bound GFP delivered using an AAV and tissue was immunolabeled for GAT-3 (blue) and SYNJ1 (red). (**B**) Astrocytes were digitally rendered (green) for isolation and quantification of co-registered signal (**C**-**D**).


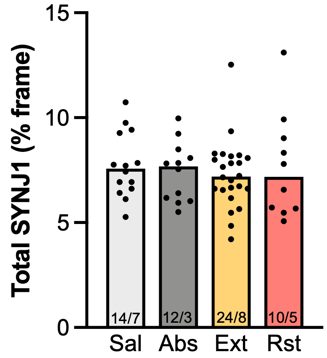


**Figure S4. SYNJ1 expression was unchanged by operant training with heroin**. (Kruskal-Wallis=0.3750 p=0.9453). Data shown as median. N shown in bars as frames/animals. Sal, yoked saline; Abs, abstinence; Ext, extinction; Rst, 15-min reinstatement.

**
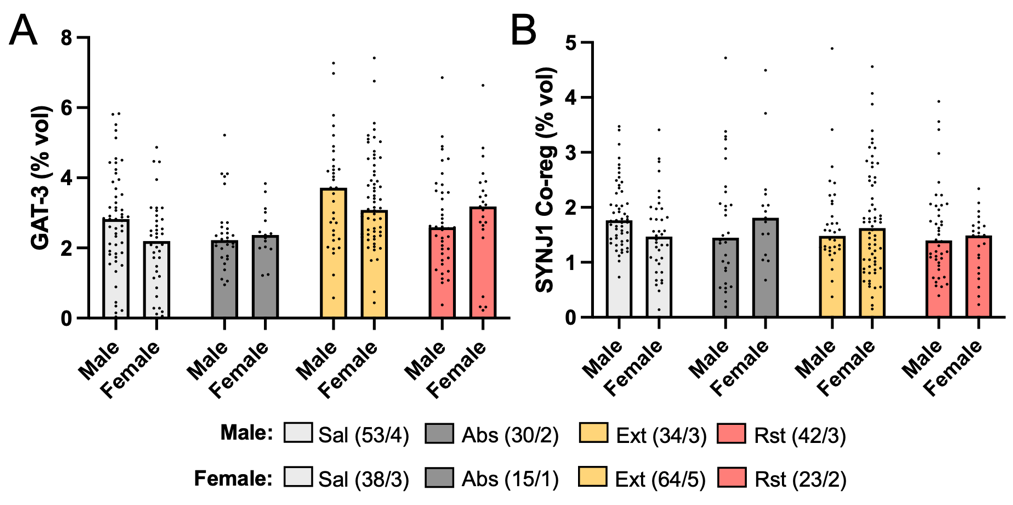
**

**Figure S5. No sex differences were observed in dlVP astrocyte measures.** GAT-3 expression (**A**, 2-way ANOVA Sex F(1,291)=2.530 p=0.1128) and SYNJ1 co-registration by dlVP astroglia (**B**, 2-way ANOVA Sex F(1,291)=0.5071 p=0.4770) were not different between male and female rats. When data were analyzed separately in males and females, GAT-3 expression was found to be elevated in both sexes (Females: Kruskal-Wallis=21.60 p<0.0001; Males: Kruskal-Wallis=13.18 p=0.0043) and SYNJ1 co-registration was found to be reduced during reinstatement in males, but not females (Females: Kruskal-Wallis=4.101 p=0.2507; Males: Kruskal-Wallis=8.821 p=0.0318, p=0.0356 Sal vs. Rst using Dunn’s test). Data shown as median. N shown in legend as cells/animals. Sal, yoked saline; Abs, abstinence; Ext, extinction; Rst, 15-min reinstatement.

**
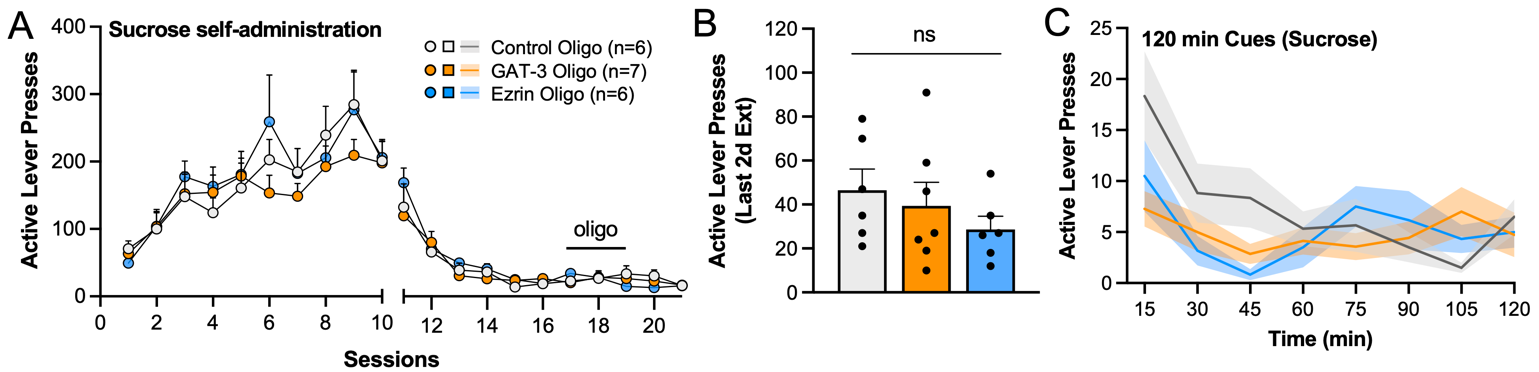
**

**Figure S6. Ezrin or GAT-3 knockdown did not impact sucrose seeking.** (**A**) Rats were trained to self-administer sucrose and received intracranial infusions of control, GAT-3 or ezrin oligo on d7-9 of extinction training. (**B**) Ezrin or GAT-3 knockdown had no impact on sucrose seeking during the last 2 days of extinction training (Welch’s ANOVA W(2,10.11)=1.272 p=0.4130) or during a 2h cued reinstatement session (**C**, 2-way ANOVA Treatment F(2,16)=1.212 p=0.3235). Animal N shown in (**A**).

**
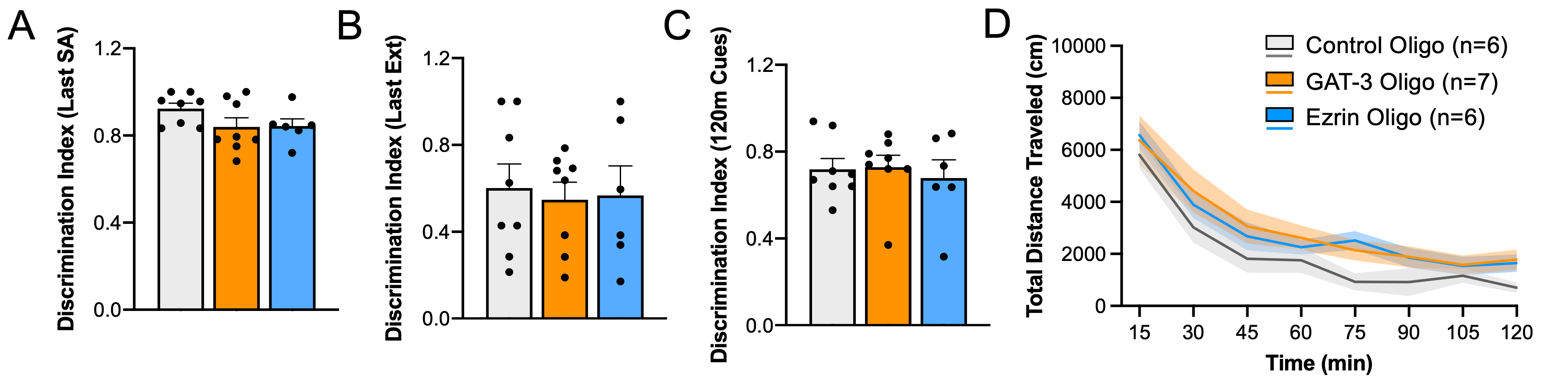
**

**Figure S7. Increased lever pressing in GAT-3 and ezrin oligo-treated rats was selective for the active lever.** A discrimination index (active/total lever presses) was calculated to determine whether increased lever pressing in oligo-treated rats reflected heroin seeking or general motor activity. A discrimination index >0.5 indicates bias for the active lever. The discrimination index was not different between animals treated with the control oligo or animals that underwent GAT-3 or ezrin knockdown during self-administration (**A**, 1-way ANOVA F(2,19)=0.7907 p=0.1672), the final extinction session (**B**, 1-way ANOVA F(2,19)=0.7457 p=0.9317), or during cued reinstatement (**C**, 1-way ANOVA F(2,19)=0.4528 p=0.8390). (**D**) Locomotor activity was unchanged by GAT-3 or ezrin knockdown in the dlVP in heroin-naïve animals (2-way ANOVA Treatment F(2,16)=1.635 p=0.2259). Data shown as mean±SEM. Animal N shown in scatter (**A**-**C**) and in legend (**D**).

**
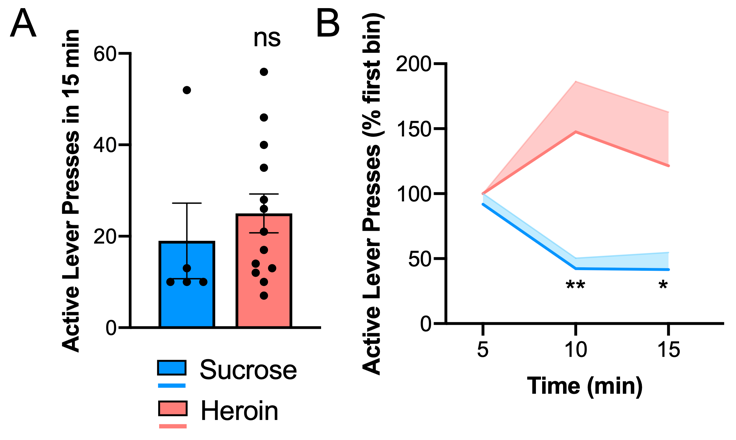
**

**Figure S8. Timecourse of reinstated heroin or sucrose seeking.** (**A**) Although lever pressing was not elevated overall during 15-min of cued heroin vs. sucrose seeking (t(16)=0.7064, p=0.4901), sucrose-trained animals underwent within-session extinction of seeking during a 15-min cued reinstatement session (**B**, 2-way ANOVA Treatment F(1,16)=3.625 p=0.0750 **p=0.0077 vs. Sucrose 5 min, *p=0.0260 v Sucrose 5 min using Tukey’s test), while heroin-trained animals did not reduce their active lever pressing during the 15-min session (**B**, p=0.4612 Heroin 5 min vs. 10 min, p=0.8630 5 min vs. 15 min using Tukey’s test). Data shown as mean±SEM.

|  |  | **Yoked** | | | | | **Abstinent** | | | | | **Extinguished** | | | | | **Reinstated** | | | | |
| --- | --- | --- | --- | --- | --- | --- | --- | --- | --- | --- | --- | --- | --- | --- | --- | --- | --- | --- | --- | --- | --- |
| **Fig** | **Variable** | **Median** | **SD** | **N** | **D'Agostino-Pearson K2** | **Normal** | **Median** | **SD** | **N** | **D'Agostino-Pearson K2** | **Normal** | **Median** | **SD** | **N** | **D'Agostino-Pearson K2** | **Normal** | **Median** | **SD** | **N** | **D'Agostino-Pearson K2** | **Normal** |
| 1 | GAT-3 | 2.508 | 1.408 | 91 | 0.807 | Yes | 2.285 | 0.906 | 45 | 9.165 | No | 3.233 | 1.704 | 98 | 65.460 | No | 2.729 | 1.821 | 65 | 50.080 | No |
|  | SYNJ1 Co-Reg | 1.658 | 0.768 | 91 | 30.580 | No | 1.534 | 1.070 | 45 | 7.051 | No | 1.562 | 0.936 | 98 | 17.810 | No | 1.472 | 0.742 | 65 | 15.310 | No |
| 2 | D1-MSN | 0.095 | 0.072 | 57 | 25.890 | No | -- | -- | -- | -- | -- | 0.152 | 0.112 | 28 | 14.300 | No | 0.103 | 0.071 | 45 | 5.780 | Yes |
|  | D2-MSN | 0.083 | 0.073 | 38 | 5.823 | Yes | -- | -- | -- | -- | -- | 0.095 | 0.075 | 26 | 21.950 | No | 0.107 | 0.101 | 36 | 11.370 | No |
| 3 | GAT-3 | 2.193 | 1.197 | 36 | 10.930 | No | -- | -- | -- | -- | -- | 2.613 | 0.892 | 37 | 3.452 | Yes | 2.932 | 1.026 | 41 | 3.423 | Yes |
|  | SYNJ1 Co-Reg | 1.285 | 0.687 | 36 | 3.761 | Yes | -- | -- | -- | -- | -- | 1.407 | 0.640 | 37 | 15.710 | No | 1.782 | 1.985 | 41 | 15.020 | No |

**Table S1. Data distribution.** Data were analyzed by Kruskal-Wallis test when populations were found not to be normally distributed using D’Agostino-Pearson normality test.

|  | **Variable** | **F** | **DFn** | **Dfd** | **P-value** |
| --- | --- | --- | --- | --- | --- |
| **Figure 1** | GAT-3 | 3.427 | 3 | 19 | 0.038 |
|  | SYNJ1 Co-Reg | 0.485 | 3 | 19 | 0.697 |
| **Figure 2** | D1-MSN | 5.804 | 2 | 10 | 0.021 |
|  | D2-MSN | 0.726 | 2 | 9 | 0.510 |
| **Figure 3** | GAT-3 | 1.629 | 2 | 12 | 0.237 |
|  | SYNJ1 Co-Reg | 2.644 | 2 | 12 | 0.112 |

**Table S2. Nested analysis confirmed main findings.** Data analysis using nested ANOVA revealed significant changes in GAT-3 expression and astroglial association with D1-MSN terminals after extinction from heroin self-administration.

|  |  |  |  |  | | | |  |
| --- | --- | --- | --- | --- | --- | --- | --- | --- |
|  |  | **Variable** | **Kruskal Wallis Statistic** | **Treatments** | **Values** | **P-value** | **η²** |  |
|  | **Figure 1** | GAT-3 | 26.65 | 4 | 299 | <0.0001 | 0.080 |  |
|  |  | SYNJ1 Co-Reg | 5.58 | 4 | 299 | 0.134 | 0.009 |  |
|  | **Figure 2** | D1-MSN | 10.38 | 3 | 130 | 0.006 | 0.066 |  |
|  |  | D2-MSN | 1.57 | 3 | 100 | 0.456 | 0.004 |  |
|  | **Figure 3** | GAT-3 | 7.70 | 3 | 114 | 0.021 | 0.051 |  |
|  |  | SYNJ1 Co-Reg | 9.32 | 3 | 114 | 0.010 | 0.066 |  |
|  |  |  |  |  |  |  |  |  |

**Table S3. Effect sizes.** An η^2^ statistic was calculated for each analysis to reflect effect size. Significant differences were observed where effect size was moderate, indicated by an η^2^>0.06^1,2^.

**References**

1 Sullivan, G. M. & Feinn, R. Using Effect Size-or Why the P Value Is Not Enough. *J Grad Med Educ* **4**, 279-282, doi:10.4300/JGME-D-12-00156.1 (2012).

2 Richardson, J. T. E. Eta squared and partial eta squared as measures of effect size in educational research. *Educational Research Review* **6**, 135-147, doi:<https://doi.org/10.1016/j.edurev.2010.12.001> (2011).
